# Long-term treatment effect and adverse events of a modified jailed-balloon technique for side branch protection in patients with coronary bifurcation lesions

**DOI:** 10.1186/s12872-018-0995-x

**Published:** 2019-01-10

**Authors:** Wenduo Zhang, Fusui Ji, Xue Yu, Xinyue Wang

**Affiliations:** Department of Cardiology, National Center of Gerontology, China, Beijing Hospital, NO.1 DaHua Road, Dong Dan, Beijing, 100730 People’s Republic of China

**Keywords:** Percutaneous coronary intervention, Modified jailed balloon technique, Coronary bifurcation lesions, Long-term

## Abstract

**Background:**

Percutaneous coronary interventions (PCI) of bifurcation lesions is technically challenging and associated with lower success rates and higher frequency of adverse outcomes. In the present study, we aimed to evaluate the immediate and long-term treatment effect and adverse events of a new modified jailed-balloon technique on side branch (SB) during PCI on coronary bifurcation lesions.

**Methods:**

This was a prospective study of 60 patients (49 males, 11 females, mean age 66 ± 10 years) with coronary bifurcation lesions treated at the Beijing Hospital between September 2014 and October 2015. They underwent main vessel (MV) stenting and modified jailed-balloon technique on the SB. All patients were followed with hospital visits at 9 months. Angiographic success, major adverse cardiac events (MACE), SB occlusion, and angina were evaluated.

**Results:**

The majority of the patients had acute coronary syndrome (91.7%) and Medina 1.1.1. bifurcation lesions (71.7%). After MV stenting, thrombolysis in myocardial infarction (TIMI) 3 flow was established 100% of MV and 93.3% of SB. No SB occlusion occurred. The jailed SB balloon and wire could be successfully removed in all patients without damage or entrapment. The majority (91.7%) of patients achieved Canadian Cardiovascular Society I stage. There was no MACE during in-hospital stay and 9-month follow-up.

**Conclusion:**

The modified JBT provided high rate of procedural success, excellent SB protection during MV stenting, and excellent immediate and long-term clinical outcomes.

## Introduction

Acute coronary syndromes (ACS) refer to a spectrum of acute myocardial ischemia and/or necrosis usually secondary to reduction in coronary blood flow and include unstable angina, non-ST-elevation myocardial infarction, and ST-elevation myocardial infarction [1, 2]. The incidence of ACS is approximately 1 million cases in the United Stated and 2 million in Europe [2]. Due to the rapid economic development of the Asia-Pacific region, including China, < 50% of the adults meet the NCEP-ATPIII low-density lipoprotein cholesterol levels [3, 4], resulting in significant mortality and morbidity [5, 6].

Atherosclerosis development involves the interplay of cardiovascular risk factors, inflammation, vascular biology and local hemodynamics [7]. Vascular geometries characterized by changes in lumen size predispose to plaque development [7]. Artery bifurcations are prone to develop atherosclerosis lesions because of the high shear stress and endothelial erosion from the turbulent blood flow [8, 9].

Approximately 15 to 20% of percutaneous coronary interventions (PCI) are performed to treat coronary bifurcation lesions [10, 11]. PCI of bifurcation lesions is technically challenging and associated with lower success rates and higher frequency of immediate and long-term adverse outcomes [12, 13]. Despite randomized studies and observational series, the selection of the optimal interventional strategy for true coronary bifurcation lesions remains controversial because of the variability in side branch (SB) disease and the desire to preserve the patency of the SB [14, 15].

According to previous studies, the one-stent strategy with provisional SB stenting is superior to the elective two-stent strategy and considered as the first option for most coronary bifurcation lesions [16, 17]. A modification of the provisional stenting strategy called the jailed-balloon technique (JBT) is designed to reduce SB occlusion during main vessel (MV) stenting, but cannot fully prevent it [18, 19].

Therefore, the aim of the present study was to explore the immediate and long-term treatment effect and adverse events of a new modified JBT for SB protection during PCI on coronary bifurcation lesions. This new technique could improve the prognosis of patients with bifurcation lesions treated with PCI.

## Materrials and methods

### Study design and patients

This was a prospective study of patients with coronary bifurcation lesions treated at the Beijing Hospital between September 2014 and October 2015. Each coronary bifurcation lesion was classified according to the Medina classification [20]. The study was approved by the Ethics Committee of Beijing hospital. Written informed consents for both the PCI procedure and participation in the study were obtained from all patients.

True bifurcation lesion, defined as a stenosis > 50% in both the MV and the ostium of the SB and Medina 1.1.1., Medina 1.0.1., Medina 0.1.1. bifurcation lesions, were included [21]. The vessel size was analyzed by quantitative coronary angiography (QCA). Patients with heavily calcified lesions, severe proximal tortuosity, cardiogenic shock, or contraindications to prolonged use of antiplatelet agents were excluded.

### Data collection

After collecting the detailed medical history and performing a complete physical examination, the baseline characteristics of the patients (including age, gender, hypertension, hypercholesterolemia, diabetes mellitus, current smoking status, and medications) were recorded.

### Pre-operative management

All patients received aspirin (300 mg) and a loading dose of clopidogrel (300 mg) prior to or at the time of selective PCI. During the procedure, an intra-arterial bolus of unfractionated heparin (UFH) was administered at 70–100 U/kg [22].

### Procedure

**Fig. 1:**
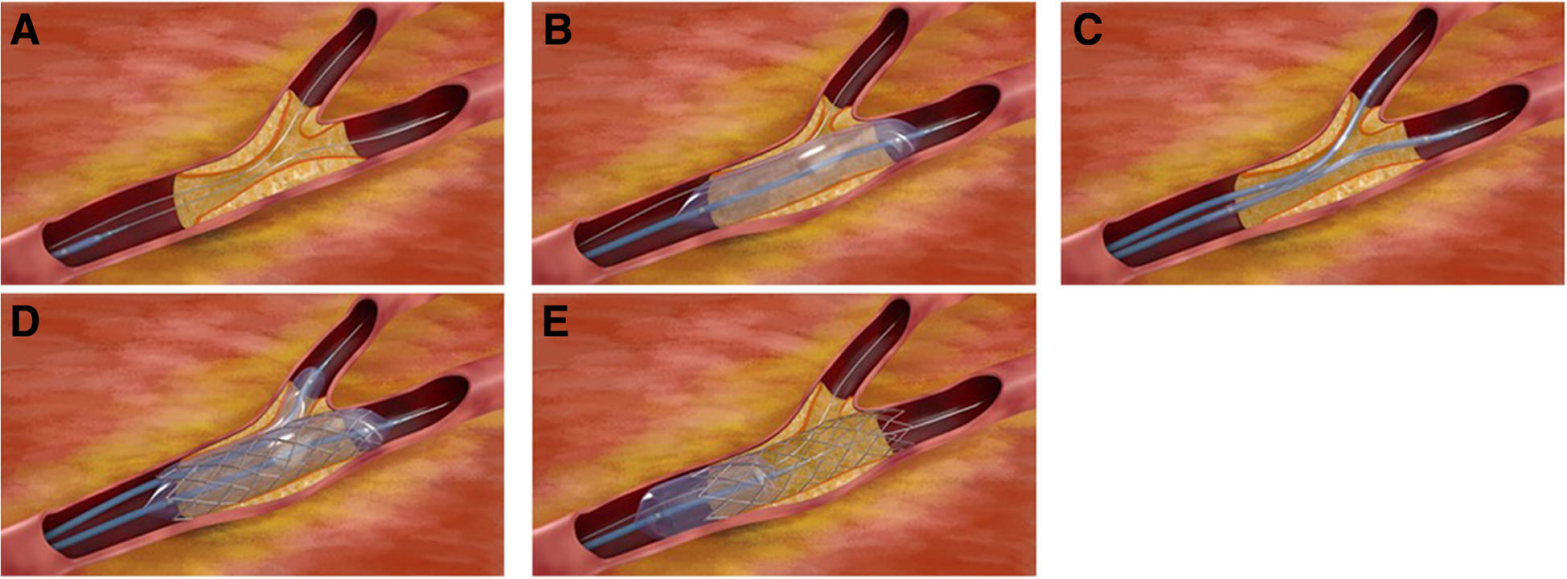
The new modified jailed-balloon technique. **a** The procedure starts with the wiring of both branches. **b** A standard POBA for the main vessel (MV) lesion. **c** The stent is sent to cover the MV lesion, then a balloon is sent into the side branch (SB). The proximal markers of the SB balloon were not beyond those of the MV stent. **d** The MV stent balloon and SB balloon were inflated simultaneously. The SB balloon was inflated to its normal pressure. Both balloon were deflated together and removed. **e** The MV stent was inflated with appropriate balloon pressures again

For the modified JBT, 6Fr guiding catheters were used via a transradial approach. The procedure is illustrated in Fig. 1. The procedure started with the wiring of both branches (Fig. 1a). The MV lesion was managed with a standard semi-compliant balloon predilatation (Fig. 1b). A stent with adequate size and length was used to cover the MV lesion, then a fitful balloon was sent into the SB; the proximal markers of the SB balloon were not beyond that of the MV stent, and the distal markers covered the SB ostium lesion (Fig. 1c). The MV stent balloon and SB balloon were inflated simultaneously; the SB balloon was inflated to the normal pressure, then both balloon were deflated together and removed (Fig. 1d). For optimization of MV stent apposition, the proximal optimization technique (POT) was performed with a short non-compliant balloon (Fig. 1e); if the SB diameter was over 2.5 mm, POT was not performed, but the wires of MV and SB were exchanged, and the procedure was completed with final kissing.

### Postoperative management

Following PCI, all patients were monitored for post-procedural complications. Cardiac troponins and creative kinase-MB were measured before the procedure and 12–18 h post-intervention. Marker elevation ≥3 times the upper limit of normal was considered significant. For patients who already had elevated cardiac enzyme levels before the procedure, marker elevation ≥50% that of the previous value was considered significant [23]. All patients were discharged following PCI with dual antiplatelet therapy (aspirin 100 mg/d and clopidogrel 75 mg/d) and followed with hospital visits for 9 months.

### Definitions and outcomes

Procedural and immediate clinical outcomes were recorded. Angiographic success was defined as successful implantation of the stent into the MV and final residual stenosis ≥30% without MV and SB flow impairment [19]. Clinical events were defined based on the recommendations of the Academic Research Consortium [23]. Major adverse cardiac events (MACE) were defined as a composite of cardiac death, myocardial infarction (MI), or target lesion revascularization (TLR). Thrombolysis in myocardial infarction (TIMI) flow grading was established for the MV and SB for each patient. SB occlusion was defined as the absence of flow in the SB immediately following MV stenting [24]. Angina was graded according to the angina classification of Canadian Cardiovascular Society (CCS) [25].

### QCA analysis

QCA analyses were performed for the MV and SB at baseline, after stent implantation, and at 9 months using the QAngio7.2 QCA software (Medis Medical Imaging System Inc., Leiden, The Netherlands). All analyses were performed in consensus by two experienced interventional cardiologists who were blinded to the patients’ characteristics. The QCA of each bifurcation lesion was obtained for the MV and SB. For quantitative analysis, at least two orthogonal projections were obtained. Angiographic frames with homogeneous contrast filling of the segment of interest were selected in a view offering good opening of the bifurcation. According to the algorithm in the dedicated software, reference vessel diameter, minimal lumen diameter, and diameter stenosis were measured in two segments.

### Statistical analysis

All calculations were performed with SPSS 17.0 (IBM, Armonk, NY, USA). Continuous variables were expressed as mean ± standard deviation (SD) and categorical variables were presented as frequency and percentage. Pre- and post-procedure QCA results were compared using the paired sample t-test or McNemar’s test, as appropriate. Two-sided *P*-values < 0.05 were considered significant.

## Results and discussion

### Characteristics of the patients

The 60 (100%) patients successfully underwent the modified JBT and were included in final analysis. The baseline clinical characteristics of the patients are shown in Table 1. The patients were 66 ± 10 years of age and 81.7% were male. Most patients (91.7%) were admitted to hospital with ACS and 58.3% patients had diabetes mellitus. Ten patients had a history of PCI.

**Table 1:** Baseline characteristics of the patients

Variable	Patients (*n* = 60)
Age (years), mean ± SD	66 ± 10
Gender, n (%)	
Male	49 (81.7)
Female	11 (18.3)
Diabetes, n (%)	35 (58.3)
Hypertension, n (%)	49 (81.7)
Smoking, n (%)	36 (45.0)
Hypercholesterolemia, n (%)	46 (76.7)
Prior PCI, n (%)	10 (16.7)
Prior by-pass surgery, n (%)	1 (1.67)
PCI indication, n (%)	
Stable angina	5 (8.3)
Non-ST acute coronary syndrome	55 (91.7)

*PCI*, percutaneous coronary intervention

### Characteristics of the lesions

Table 2 shows that all the patients were operated using the transradial approach and six Fr guiding catheters. The lesions were in the distal left main (LM) coronary artery in eight patients (13.3%), the left anterior descending artery (LAD)-diagonal branch (D) level in 48 (80.0%), the left circumflex artery (LCX)-obtuse marginal branch (OM) level in one (1.7%), and the right coronary artery (RCA) posterior descending artery (PD)-posterolateral artery (PL) level in three (5.0%). Forty-three (71.7%) patients had Medina type 1.1.1 lesion. Rates of pretreatment TIMI 3 flow in MV and SB were in 100 and 78.3%, respectively. Predilatation of MV and SB was performed in 100 and 0% lesions, respectively. All implanted stents were second-generation drug eluting stent.

**Table 2:** Angiographic and procedural characteristics

Variable	Patients (n = 60)
Lesion location, n (%)	
LM\LAD\LCX	8 (13.3)
LAD\D	48 (80.0)
LCX\OM	1 (1.7)
RCA\PD or PL	3 (5.0)
Medina type, n (%)	
1.1.1	43 (71.7)
1.0.1	7 (11.7)
0.1.1	10 (16.7)
MV pretreatment TIMI flow, n (%)	
0–1	0
2	0
3	60 (100)
SB pretreatment TIMI flow, n (%)	
0–1	1 (1.7)
2	12 (20.0)
3	47 (78.3)
Transradial approach, n (%)	60 (100)
Predilatation, n (%)	
MV	60 (100)
SB	0
MV size (mm), mean ± SD	
Diameter	3.18 ± 0.44
Length	22.52 ± 7.82
SB size (mm), mean ± SD	
Diameter	2.11 ± 0.41
Length	10.03 ± 4.41

*LM*, left main coronary artery; *LAD*, left anterior descending artery; *D*, diagonal branch; *LCX*, left circumflex branch; *OM*, obtuse marginal branch; *RCA*, right coronary artery; *PD*, posterior descending artery; *PL*, posterolateral branch; *SB*, side branch; *MV*, main vessel

### Outcomes

Immediate procedural, post-procedural, and 9-month outcomes are shown in Table 3. The procedural success rate was 100%. After MV stenting, there was no SB loss and all patients had TIMI 3 flow in the MV. In only one (1.7%) patient the SB remained TIMI 1 flow compared with pre-procedure. Three patients were with TIMI 2 flow due to long- and high-grade stenosis at SB ostium after MV stenting. After giving nitroglycerin in the coronary artery, coronary flow reached TIMI 3 flow in two patients. Therefore, the final kissing balloon inflation was performed in only one patient. No patient needed additional stent due to proximal or distal stent edge dissection. The jailed SB balloon and wire could be successfully removed in all patients without damage or entrapment. The peri-procedural MI rate was 0% and there was no MACE during in-hospital stay and 9-month follow-up. The majority (91.7%) of patients achieved CCS I stage. No patient’s symptoms exacerbated to CCS III-IV stage.

**Table 3:** Initial procedural and clinical outcomes

Variable	Post-procedur (n = 60)	After 9 months (n = 60)
Procedural success, n (%)	60 (100)	NA
Peri-procedural MI, n (%)	0	0
SB loss, n (%)	0	0
MV TIMI flow after MV stenting, n (%)		
0	0	0
1	0	0
2	0	0
3	60 (100)	60 (100)
SB TIMI flow after MV stenting, n (%)		
0	0	0
1	1 (1.7)	0
2	3 (5.0)	0
3	56 (93.3)	60 (100)
SB dissection, n (%)	0	0
SB stenting, n (%)	0	0
Final kissing balloon inflation, n (%)	1 (1.7)	NA
Death, n (%)	0	0
MI, n (%)	0	0
Repeat PCI or CABG, n (%)	0	0
CCS, n (%)		
I	NA	55 (91.7)
II	NA	5 (8.3)
III-IV	NA	0

*SB*, side branch; *TIMI*, thrombolysis in myocardial infarction; *MV*, main vessel; *MI*, myocardial infarction; *NA*, not applicable; *PCI*, percutaneous coronary intervention; *CABG*, coronary artery bypass graft; *CCS*, Canadian Cardiovascular Society

### QCA

QCA results for both the MV and SB at baseline, after procedure, and after 9 months are shown in Table 4. The mean post-procedure minimum lumen diameter in MV and SB were 2.79 ± 0.45 mm and 0.84 ± 0.59 mm, respectively. Compared with baseline, there was significant difference in post-procedure and after 9 months in MV (*P* < 0.001). Although only 93.3% patients achieved TIMI 3 flow post-procedure, all patients achieved TIMI 3 flow at 9 months (P < 0.001 vs. post-procedure).

**Table 4:** Quantitative coronary angiographic analysis

Variable	Baseline (n = 60)	Post procedure (n = 60)	After 9 months (n = 60)
Main vessel, mean ± SD			
MLD (mm)	1.16 ± 0.41	2.79 ± 0.45^&^	2.67 ± 0.32^&^
Diameter stenosis (%)	50.98 ± 12.97	12.06 ± 6.09^&^	10.62 ± 6.767^&^
Side branch, mean ± SD			
MLD (mm)	0.83 ± 0.59	0.84 ± 0.59	0.83 ± 0.67
Diameter stenosis (%)	68.32 ± 21.02	47.94 ± 23.82	63.04 ± 28.23
TIMI flow 3, n (%)	47 (78.3)	56 (93.3)^&^	60 (100)^&#^

*MLD*, minimum lumen diameter; *TIMI*, thrombolysis in myocardial infarction;

^&^*P* < 0.05 vs. baseline

^#^P < 0.05 vs. post-procedure

## Results and discussion

PCI of bifurcation lesions is technically challenging and associated with lower success rates and higher frequency of adverse outcomes. The objective of the present study was to evaluate the immediate and long-term treatment effect and adverse events of a new modified jailed-balloon technique on side branch (SB) during PCI on coronary bifurcation lesions. The results suggest that the modified JBT provided high rate of procedural success, excellent SB protection during MV stenting, and excellent immediate and long-term clinical outcomes.

In our study, the majority of patients presented with ACS (91.7%) and 71.7% bifurcation lesion were Medina type 1.1.1. All 60 (100%) patients successfully underwent this modified JBT. Although there were high clinical and angiographic risks for SB occlusion during MV stenting, we did not observe any SB occlusion post-procedure. Indeed, the advantage of this technique is that acute occlusion of SB is very unlikely to happen because the SB balloon is expanded while the stent is inflated, so that the blood vessels on the SB will not show a snow shoveling effect, and the ostium will not be occluded. Hence, the peri-procedural MI rate was 0% and there was no MACE during in-hospital stay and 9-month follow-up, and most (91.7%) patients achieved CCS I stage. Depta et al. [26] showed that JBT was associated with a significantly lower rate of MACE compared with no JBT. Therefore, we think that this modified JBT could be better than JBT.

Previously, the jailed guidewire technique has been proven to be effective during the provisional technique [27], and it is nowadays widely adopted in the clinical practice but is also associated with the risk of jailed wire entrapment and does not abolish the risk of SB occlusion. Furthermore, there are several reports of severe complications caused by guidewire fracturing during withdrawal [28]. Burzotta et al. [19] developed the “jailed-balloon technique,” a modified provisional method. In this new method, SB was lost after MV stenting in 15% of patients. Furthermore, they had to implant another stent to SB due to suboptimal outcomes in 50% of patients. Therefore, the risk of SB occlusion is present after MV stenting due to plaque shift into the SB [29]. Compaired with original JBT of Burzotta et al.,which SB balloon are only semi-inflated, the main difference of our modified JBT is that SB balloon was inflated to fully normal pressure. By this new modified JBT, the SB ostium stenosis could be adequately reshaped and no patient suffered from TIMI flow 0 in SB.

**Fig. 2:**
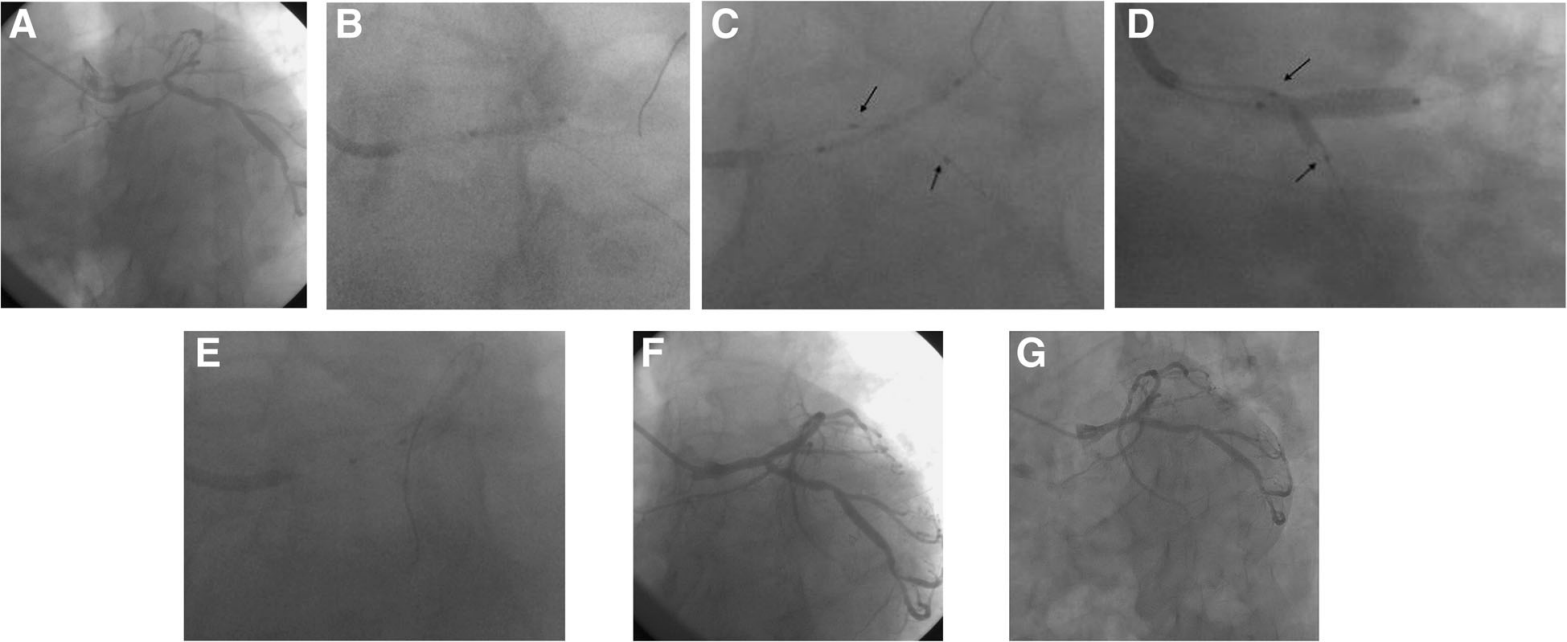
**a** Lesion angiography before procedure; (**b**) The MV lesion was managed with a standard semi-compliant balloon predilatation; (**c**)A stent with adequate size and length was used to cover the MV lesion, then a fitful balloon was sent into the SB; the proximal markers of the SB balloon were not beyond that of the MV stent(Black Arrow), and the distal markers covered the SB ostium lesion(Black Arrow); (**d**)The MV stent balloon and SB balloon(Black Arrow)were inflated simultaneously; the SB balloon was inflated to the normal pressure; (**e**) For optimization of MV stent apposition, the proximal optimization technique (POT) was performed with a short non-compliant balloon; (**f**)Immediate angiography after operation;(**g**) 9 months later angiography after operation

The major issues about the modified JBT are the possible risk of MV stent struts distortion/malapposition in the MV proximal segment, entrapment of the SB balloon under the MV stent, and SB ostial dissection. As described in the Methods, the proximal markers of the SB balloon were not beyond that of the MV stent, and the distal markers covered the SB ostium lesion. Because of this, the jailed SB balloon was removed successfully in all patients without damage or entrapment of the balloon, as observed in previous studies [19, 30]. Because the SB balloon is inflated to its nominal pressure, SB ostium stenosis could be adequately reshaped, as in a study by Cayli et al. [31], and no patient suffered from TIMI flow 0 in SB. Good outcomes were also observed in a Japanese study using a modified JBT that is slightly different from ours in the choice of stents [32]. And the good long-term effect was observed in the imaging of many patients in our study and SB Balloon markers(Black arrow) was pointed (Fig. 2).

Although SB TIMI 1 was observed in one patient (1.7%) and TIMI 2 in three (5%) patients. In the four cases’ with SB blood low flow, the diameter of SB was 2.5–3.0 mm. According to our research experience, SB slow flow happened due to lesion type all Medina 1.1.1,and the serious stenosis(more than 90%),not by diameter and length of the SB. However, Those patients did not need another stent to SB and recovered to TIMI 3 in SB at 9 months. That is advantage of this modified JBT technology. By this new modified JBT, the SB ostium stenosis could be adequately reshaped, and it might cause no flow fo more dissection happen by two times dilation of SB. On the other hand, proximal MV stent shape was well inflated. After both inflations, if SB >2.5 mm, for optimization of MV stent apposition, the POT was routinely performed with a short non-compliant balloon after removing the jailed balloon. Due to POT, MV stents were well expanded in all patients of the present study. Compared with baseline, the MLD was significantly different after the procedure and after 9 months. All PCI in the present study were performed using 6Fr-guiding catheters via a transradial approach, resulting in less pain after the procedure.

The present study has some limitations. First, the sample size was relatively small. Secondly, the study population was relatively homogenous, characterized by Chinese adults, middle- and old-aged, presenting at a single health Institute. Thirdly, this technique was not compared with other techniques such as traditional provisional technique and JBT. Fourthly, the follow-up was short. Finally, intravascular ultrasound (IVUS) or optical coherence tomography (OCT) were not used in our study. Nevertheless, the present study provides preliminary data for future multicenter randomized controlled trials.

## Conclusions

This modified JBT showed a high rate of procedural success and excellent SB protection during PCI of coronary bifurcation lesions. The treatment effect was good and there were no adverse events immediately after the procedure and during the 9-month follow-up.

## Abbreviations

ACS: Acute coronary syndromes
CCS: Canadian Cardiovascular Society
IVUS: Intravascular ultrasound
JBT: Jailed-balloon technique
LAD: Left anterior descending artery
LCX: Left circumflex artery
LM: Left main
MACE: Major adverse cardiac events
MI: Myocardial infarction
MV: Main vessel
OCT: Optical coherence tomography
PCI: Percutaneous coronary interventions
PD: Posterior descending artery
PL: Posterolateral artery
POT: Proximal optimization technique
RCA: Right coronary artery
SB: Side branch
TIMI: Thrombolysis in myocardial infarction
TLR: Target lesion revascularization
UFH: Unfractionated heparin
